# Reconstructing the pulmonary niche with stem cells: a lung story

**DOI:** 10.1186/s13287-022-02830-2

**Published:** 2022-04-11

**Authors:** Barbie Varghese, Zihan Ling, Xi Ren

**Affiliations:** Department of Biomedical Engineering, Carnegie Mellon University, Scott Hall 4N111, 5000 Forbes Avenue, Pittsburgh, PA 15213 USA

## Abstract

The global burden of pulmonary disease highlights an overwhelming need in improving our understanding of lung development, disease, and treatment. It also calls for further advances in our ability to engineer the pulmonary system at cellular and tissue levels. The discovery of human pluripotent stem cells (hPSCs) offsets the relative inaccessibility of human lungs for studying developmental programs and disease mechanisms, all the while offering a potential source of cells and tissue for regenerative interventions. This review offers a perspective on where the lung stem cell field stands in terms of accomplishing these ambitious goals. We will trace the known stages and pathways involved in in vivo lung development and how they inspire the directed differentiation of stem and progenitor cells in vitro*.* We will also recap the efforts made to date to recapitulate the lung stem cell niche in vitro via engineered cell–cell and cell-extracellular matrix (ECM) interactions.

## Introduction

The mammalian respiratory system is a concert between two very elaborate branching structures: the pulmonary epithelium which transports air to the alveoli, and the vasculature which carries blood through the alveoli for gas exchange [1]. The pulmonary epithelium is continually lined with distinct populations of progenitor and specialized cells whose functions are defined by their location along the branched network. For instance, the proximal lung, comprising the trachea and bronchi, expresses a pseudostratified epithelium including basal cells that are the stem cells of the airway, goblet cells that secrete mucus, ciliated cells that move mucus in a cephalic direction along the airway, and neuroendocrine cells that serve as airway sensors [1, 2]. In comparison, 95% of the distal lung is lined with thin, elongated type 1 alveolar epithelial cells (AEC1) that facilitate gas exchange. The remaining 5% of alveolar surface is dominated by surfactant-producing type 2 alveolar epithelial cells (AEC2) [3]. Additionally, the lung epithelium is surrounded by a mesenchymal compartment that comprises a wide variety of cell types such as fibroblasts, endothelial cells, and smooth muscle cells.

Lung organogenesis is a highly elaborate and coordinated process. Most of what we know about the developing human lung comes from studying rodent models. However, there are apparent discrepancies between human versus rodent lung development. For example, alveolarization is initiated only after birth in rodent lungs [4, 5]. In contrast, alveolar maturation begins prior to birth in human lungs [1]. There are also differences in the population and location of certain stem and progenitor cells in the lungs of both species [1]. For instance, basal cells line the entire conducting airway in humans, but are restricted to the trachea and main stem bronchi in mice [6, 7]. Additionally, the alveoli and bronchioles in mouse lungs maintain a population of bronchio-alveolar stem cells (BASCs) that can give rise to both proximal and distal lung epithelial cell types [5, 8–10]. However, the presence of BASCs in human lungs remains uncertain [11]. As will be revealed later, there are also inherent differences in the genes and signaling pathways involved in the temporal specific regulation of lung development among both species [12–14].

Thus, there is a gaping need for in vitro platforms that can closely recapitulate the complexity of human lung development and physiology with improved fidelity. Embryonic stem cells (ESCs) and induced pluripotent stem cells (iPSCs), collectively referred to as human pluripotent stem cells (hPSCs) provide a unique opportunity to apply the developmental signaling mechanisms understood from native embryogenesis to recapitulate key aspects of human lung development that were previously unfeasible to study [1]. While ESCs have provided much needed insight into the derivation of lung cells, their use toward clinical research and application is challenged by limited availability and ethical concerns [15]. The use of iPSCs, that can be induced to a pluripotent status from somatic cells not only offsets these limitations, but also realizes the promise of personalized medicine as such cell lines can be generated from any individual [16]. Personalized disease modeling and drug screening immediately comes to mind as some of the potential outcomes of iPSC research. Currently, hPSC-derived lung epithelial cells possess an immature phenotype unfit for transplantation, leaving many areas of active investigation [17, 18]. Generating cells and eventually, tissues and organs for clinical use remains the crown jewel of the lung field.

## In vitro recapitulation of the developmental program of the lung

Prior studies have characterized the adult lung as a relatively quiescent organ with extremely low levels of cellular turnover and a limited capacity for regeneration [19]. Only recently has this slow but continuous renewal of lung tissue by endogenous stem and progenitor cells garnered interest and appreciation [20]. Several attempts have been made to recapitulate lung development via in vitro hPSC differentiation; gradually progressing from inducing progenitor stages such as definitive endoderm [21–24], and anterior foregut endoderm [25, 26] to deriving lung-specific epithelial progenitors [14, 27, 28] and further to specialized respiratory epithelial cell types [29–34]. Provided below is an up-to-date description of the differentiation process as inspired by the in vivo developmental program of the murine lung.

### Definitive endoderm

**Fig. 1:**
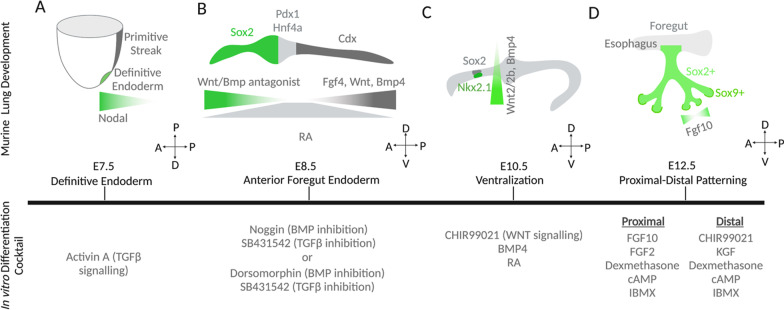
Directing lung stem cell differentiation in vitro via introduction of signaling ligands or small-molecule regulators mimicking in vivo lung development. **a** In mid-gastrula stage, DE elongates from the anterior end of primitive streak. The Nodal^high^ region gives rise to the future foregut. **b** The AP axis along the gut tube is established at the end of gastrulation, regulated by the molecular gradient of ligands and antagonists of Wnt, Fgf4, and Bmp signaling. **c** The ventralization of foregut is regulated by the gradient of Wnt2/2b and Bmp, and its completion is marked by the emergence of Nkx2.1-positive lung progenitor cells. **d** The proximal–distal patterning and branching morphogenesis of developing lung is guided by a lateral inhibition mechanism of Fgf10. A/P, anterior/posterior. P/D, proximal/distal. D/V, dorsal/ventral

The lung epithelium, purely of endodermal origin, emerges from a small population of Nkx2.1 positive progenitor cells. Canonical Wnt/ β-catenin signaling and Transforming Growth Factor-β (TGF-β) signaling via Nodal drives the initial specification of primitive streak and then endodermal lineage in the embryo; cells that ingress through the primitive streak experiencing greater Nodal signaling are specified as definitive endoderm (DE) (Fig. 1a) [22]. Accordingly, DE induction is also the first act in hPSC differentiation into specialized pulmonary epithelial cells in vitro [35]. Activin A, a nodal protein alternative is commonly used to specify DE in in vitro stem cell differentiation [23, 24, 36].

### Anterior–posterior (AP) patterning of the gut tube

After gastrulation (E7.5-9.0 in mice), the DE folds to form a primitive gut tube that further differentiates along the AP axis into several organ-specific epithelial lineages that eventually give rise to most organs in the digestive system and the lung [37]. Endodermal patterning is inherently driven by the programmed crosstalk between the DE and its surrounding mesenchyme via Wnt, Bone morphogenetic protein 4 (Bmp4), retinoid acid (RA) and Fibroblast growth factor 4 (Fgf4) signaling, resulting in the Sox2^high^ anterior foregut, Pdx1^high^ posterior foregut, and Cdx^high^ hindgut at E8.5 of mouse embryogenesis (Fig. 1b) [38–41]. Specifically, the esophagus, trachea, stomach, lungs, thyroid, liver, and pancreas are derived from the foregut, while the small and large intestines are derived from the midgut and hindgut, respectively [38].

Within the foregut, the lung is further specified in the anterior portion, known as the anterior foregut endoderm (AFE). Accordingly, deriving AFE cells from DE marks the next stage in hPSC differentiation into lung epithelium. Established cocktails for in vitro DE anteriorization usually include a combination of BMP and TGF-β inhibition. Some known examples include the combined use of NOGGIN (BMP inhibitor) and SB431542 (TGF-β inhibitor) or Dorsomorphin (BMP inhibitor) and SB431542 (TGF-β inhibitor) [25, 27].

### Dorsal–ventral (DV) patterning of the AFE

Signals from the surrounding mesenchyme also establish a gradient along the DV axis of the AFE [42]. During this time in development (E9.0 in mice), one of the earliest markers of the lung epithelium, Nkx2.1 is specified in a small group of cells located along the ventral side of the AFE, marking the start of the embryonic phase of lung development (spanning up to E12.5 in mice) [43–46]. Although the emergence of lung progenitors is marked by the induction of Nkx2.1 expression, the deletion of Nkx2.1 gene in mice leads to abnormal but continued lung development [47].

Between E9.0-E10.5 in mice, the mesenchyme which surrounds the AFE exhibits a gradient of β-catenin activated by Wnt2/2b ligands concentrated at the ventral side of the foregut [40, 45, 48–50]. A Wnt2/2b double knockout model in mouse embryos was found to be devoid of any Nkx2.1 expression or lung organogenesis, echoing the importance of the contributions made by β-catenin signaling during lung specification in the foregut endoderm [45, 51]. Additionally, at E9.0 in mice, BMP signaling from the mesenchyme surrounding the ventral foregut suppresses Sox2 (indicative of esophageal fate) in the ventral endoderm, further allowing for the specification of respiratory fate via Nkx2.1 expression. (52) In essence, the gradient established by Wnt2/2b and Bmp4 defines the dorsal Sox2^high^ esophageal region and the ventral Nkx2.1^high^ lung territory (Fig. 1c) [41, 45, 52–54].

At E10.5 in mice, the Nkx2.1 positive progenitor cells evaginate to initiate lung budding at the ventral wall of the AFE. This is facilitated by Fgf10 signaling from the surrounding mesenchyme [55]. Besides Fgf10, RA signaling also plays a crucial role in lung bud formation as it promotes Wnt2/2b signaling by suppressing the Wnt inhibitor Dickkopf-related protein 1 (Dkk1); this is vital for conserving Nkx2.1 identity among the progenitors [56]. Additionally, RA signaling suppresses TGF-β signaling in the surrounding mesenchyme, which is critical for Fgf10 mediated lung budding [57].

Accordingly, ventralization of AFE cells to generate NKX2.1-positive lung progenitors is the next logical step in directed lung epithelial differentiation from hPSCs. Most ventralization cocktails utilize some combinations of developmentally inspired signaling molecules such as BMP4, FGF10, Keratinocyte Growth Factor (KGF), RA, and WNT activation via CHIR99021 (a GSK3β inhibitor) [58]. A recent study found that FGF signaling was dispensable for NKX2.1 specification during the ventralization stage, and established three essential factors for NKX2.1 induction, CHIR99021 (Wnt agonist via GSK3β inhibition), BMP4 and RA [59]. Further, during the codifferentiation of cardiac and pulmonary lineages, effective NKX2.1-positive lung progenitor induction can be obtained by adding only CHIR99021 and RA during ventralization [60].

### Proximal–Distal patterning and branching morphogenesis

**Fig. 2:**
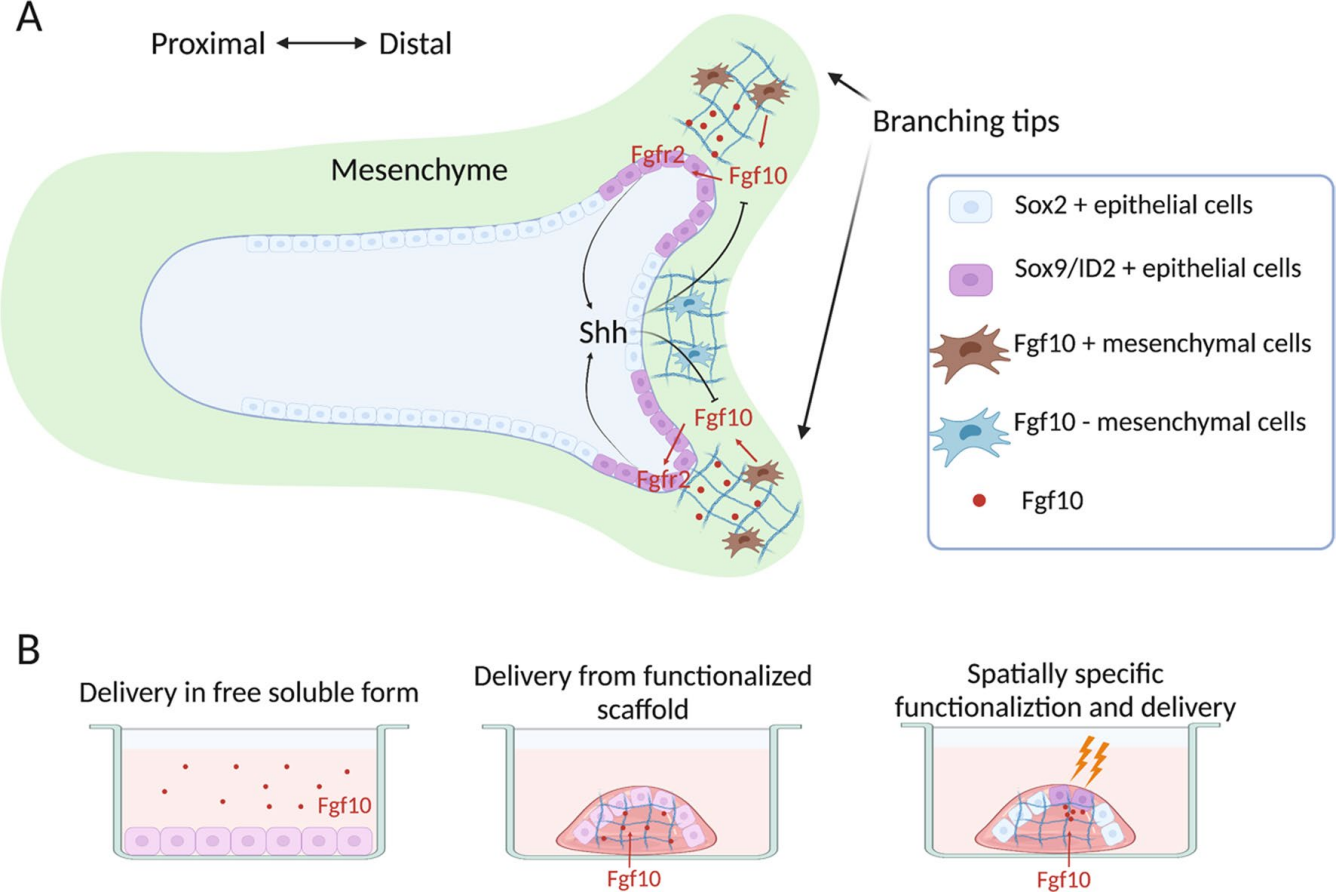
Recapitulating the lung cell-ECM interaction in vitro. **a** Cell-ECM interaction during in vivo branching morphogenesis. Fgf10 in the mesenchyme surrounding the branching tips specifies the Sox9/ID2 positive cell fate. **b** Schematics showing how engineered ECM can modulate cellular interactions with key morphogenic growth factors (such as FGF10) in human lung stem cell engineering. Traditional FGF10 is delivered in its free soluble form. Using functionalized ECM with enhanced affinity to FGF10, such as that modified with heparin, more durable bioactivity and biomimetic ECM association can be introduced. Further, spatial specific delivery or activation of growth factors, using approaches such as photo-activatable biomaterials, can offer further control over directed tissue formation, mimicking the mechanism underlying native branching morphogenesis. P/D, proximal/distal

During the pseudoglandular stage of lung organogenesis (E12.5-E16.5 in mice), the lung buds that sprout from the ventral foregut endoderm elongate and branch into the surrounding mesenchyme via a process called branching morphogenesis. Specifically, the distal epithelial tips undergo a period of repetitive bifurcations that results in the highly arborized tissue network that is characteristic of the lung [43, 61]. As hinted earlier, Fgf10 plays a critical, however, distinct role in branching morphogenesis of mouse and human lungs. In mice, Fgf10 from the distal lung mesenchyme surrounding the branching epithelial tips acts on Fgfr2-expressing epithelial cells to promote the expression of Bmp4 and Shh (Fig. 2a), which in turn inhibits Fgf10 expression. This lateral inhibition mechanism drives the outgrowth of new epithelial branches [62, 63]. In contrast, the role of FGF10 in human branching morphogenesis remains unclear. In human fetal lung explant culture, FGF10 treatment decreased the number of SOX2/SOX9 double positive cells and failed to induce branching [64, 65]. Another study found that the removal of FGF10 from in vitro bud tip progenitor culture did not affect the expression of human distal tip markers such as SOX2 and SOX9 [14].

At the same time (in mice), the Nkx2.1-positive progenitors lining the epithelium start to exhibit distinct fate along the proximal–distal axis, resulting in Sox2-positive proximal lung progenitors and Sox9/Id2-positive distal lung progenitors. Interestingly, Sox2 expression makes multiple comebacks over the course of fetal lung development: first as a pluripotency marker during gastrulation, then during anteriorization of the foregut endoderm, and finally during branching morphogenesis at the primary bronchial stalk in basal cells alongside P63. In fact, this final-stage Sox2 expression is maintained throughout the conducting airway morphogenesis until adulthood, suggesting the importance of Sox2 in driving epithelial differentiation in the airway, including into secretory cells, ciliated cells, and secretory cells [54, 66–68]. In the meantime, Sox9/Id2-positive cells specified in the distal lung eventually differentiate into AEC1 and AEC2 at the branching tips (Fig. 1d) [20, 69]. This marks yet another difference between mouse and human lungs, as during the pseudoglandular stage, human distal lung tips are double positive for both SOX2 and SOX9. However, this SOX2/SOX9 double positive feature is later lost during the canalicular stage of human lung development as these progenitor cells further differentiate [12].

The canalicular stage (E16.5-E17.5 in mice) marks the emergence of the pulmonary parenchyma and the air-blood barrier. During this stage, the terminal lung buds flatten and a thin air-blood barrier appears where the capillaries directly contact the flattened epithelium comprising AEC1 and AEC2 pneumocytes [43]. Eventually, during the saccular stage (E17.5-P0 in mice), these terminal buds develop into small sacs acting as alveolar precursors. At the same time, AEC2 starts to produce respiratory surfactants. While surfactants are initially stored in the intracellular lamellar bodies of AEC2, it is later secreted into the alveoli to reduce surface tension at the air–liquid interface.

Finally, just prior to birth, the developing lung enters the alveolar stage, lasting well into early childhood in both mice and human. Here, the small alveolar sacs formed during the canalicular, and saccular stages undergo further division via secondary septation to increase the surface area available for gas exchange [70]. Additionally, the capillary units associated with the alveoli become tightly apposed to AEC1 for enhanced gas-exchange efficiency [71]. Alveologenesis and vasculogenesis continues until the lungs enter quiescent homeostasis.

A few protocols have already established the derivation of both proximal and distal lung cells together from hPSC-derived NKX2.1 positive progenitors. Known recipes include a combination of CHIR99021, FGF10 and KGF, and a combination of CHIR99021, FGF10, KGF, BMP4 and RA [27, 30]. These protocols often yield a combination of multiple cell types, rendering them extremely useful as models for development. Below is a discussion of existing protocols for the directed differentiation of hPSC-derived NKX2.1 positive progenitors to specific pulmonary epithelial lineages.

- Basal cells: A recent study implicated Wnt signaling as the driver of the bifurcation between proximal (low Wnt) versus distal (high Wnt) fate among Nkx2.1-positive lung progenitors [72]. FGF10 signaling has been shown to maintain epithelial progenitors in an undifferentiated state [73]. It is also highly expressed throughout the parenchyma in the human airway and resident vascular smooth muscle cells [64, 65]. Accordingly, a medium without exogenous Wnt, and with FGF10 and FGF2 was developed for deriving airway basal cells from hPSCs [32]. 3D Matrigel culture was then initiated in a medium comprising FGF2 and FGF10, together with Dexmethasone, Cyclic adenosine monophosphate (cAMP) and 3-Isobutyl-1-methylxanthine (DCI). The resulting cells were marked by the co-expression of the typical basal cell markers such as NKX2.1, P63, and SOX2, and are capable of long-term self-renewal and differentiation into ciliated and secretory cells in a way similar to what primary basal cells do.
- AEC2: AEC2s serve several functions in the lung, including secreting surfactants to maintain the patency of the alveoli, and further differentiating into AEC1s [74]. The differentiation of hPSC-derived AEC2s capable of long-term in vitro self-renewal was recently reported [31]. NKX2.1-positive lung progenitors, enriched by fluorescence activated cell sorting (FACS), were transferred to 3D Matrigel-embedded culture in the presence of CHIR99021 (WNT agonist), to promote distal lung specification. KGF was also supplemented to the medium, due to its role in inducing AEC2 differentiation and proliferation in vivo [75]. Additionally, cAMP and 3-Isobutyl-1-methylxanthine (cAMP signaling agonist) were also added to promote alveolar maturation [76]. Following two weeks of treatment with the CK-DCI (CHIR99021, KGF and DCI) medium, CHIR99021 (Wnt) exposure was withdrawn for one week, followed by CHIR99021 addback. The resulting protocol was able to successfully enable the formation of alveolospheres comprising SFTPC-positive AEC2s. The withdrawal and subsequent addback of CHIR99021 is consistent with the Wnt signaling wave observed during murine lung development and is required for AEC2 maturation and long-term self-renewal [77]. The induced AEC2s also possessed functional lamellar bodies and were capable of surfactant secretion, a key function of AEC2 in lung physiology. However, the SFTPC-positive hPSC-derived AEC2s generally possessed an immature phenotype in comparison with their in vivo counterpart, and additional molecular signatures, such as ABCA3, are likely required to define mature AEC2s [78, 79].
- AEC1: AEC1s facilitate the primary function of the lung – gas exchange. Historical evidence suggests that AEC2s isolated from rats that are seeded on tissue culture-treated plastic readily transdifferentiate into AEC1-like cells, expressing similar morphology and markers as native AEC1s [80]. More recently, a serum-free, feeder-free protocol was developed to differentiate primary human AEC2 to AEC1 in vitro [81]. This media combined SB431542 (TGF-β inhibitor), CHIR99021 (Wnt agonist), BIRB796 (that inhibits mitogen-activated protein kinase or MAPK), FGF10, and Epidermal Growth Factor (EGF) in the presence of human serum. The resulting cells were marked by an increase in AGER (AEC1 marker) and a decrease in SFTPC expression. These cells also possessed a flat, thin morphology characteristic of AEC1. Additionally, hPSC-derived AEC2s cultured in 2D conditions possessed flat AEC1-like morphology, along with the upregulation of AEC1 markers such as AGER, CAV1 and PDPN [31]. Similar AEC1-like cells were also observed in an hPSC-derived lung bud organoid model [82]. More recently, hPSC-derived AEC2s co-cultured with fetal lung fibroblasts differentiated into alveolar organoids comprising both AEC1s and AEC2s [33]. They also demonstrated that the AEC2s further differentiate into AEC1s in the presence of XAV-939 (Wnt inhibition), possessing AEC1-like flat and thin cell morphology, in addition to expressing a combination of AEC1 markers, including AGER, HT1-56, PDPN, and HOPX. Single cell RNA sequencing also revealed considerable similarities to primary AEC1s. Despite progresses discussed here, the mechanisms driving hPSC differentiation into AEC1s remain not completely understood and functional assays for fully characterizing the induced AEC1s remains limited, which presents opportunities for future investigation.

## Pulmonary organoids: organized cell–cell interactions

The human lung is home to over 40 individual cell types that coordinate to maintain respiratory function [83, 84]. Accordingly, communication between these participating cells is often at the heart of major events such as lung organogenesis and pathogenesis. However, this communication maybe lost in translation going from three-dimensional (3D) organs to two-dimensional (2D) cell culture models, citing the need for 3D tissue platforms that can recreate the native-like tissue microenvironment in vitro. Lung organoids are 3D micrometer-to-millimeter-scale tissues generated from primary or hPSC-derived epithelial progenitors that self-assemble and further differentiate to recapitulate some aspect(s) of respiratory tissue architecture and physiology in an in vitro setting. They can be broadly classified as: proximal lung organoids that feature cells mimicking the conducting airway, distal lung organoids that recapitulate the alveoli, or proximal–distal organoids.

### Proximal lung organoids

While the conducting airway generally exhibits slow cellular turnover, it has its own population of adult stem cells, the basal cells, which can renew and replenish the airway epithelium in the event of an injury [85]. Airway epithelial cells are relatively easy to acquire from patients given that they can be isolated from nasal swab cultures [86]. As discussed earlier, basal cells can also be generated from hPSCs [72, 87, 88]. Such easy access has opened up the possibility of generating airway disease models that leverage the knowledge of in vivo differentiation cues to drive basal cell specification toward a muco-ciliary fate in vitro.

The in vitro self-organization of airway epithelial cells in collagen into 3D tubular structures comprising basal, secretory, and ciliated cells was first reported almost 30 years ago [89]. Almost two decades later, the first account of basal cell self-organization and differentiation into airway organoids was reported. Using air–liquid interface (ALI) culture and Matrigel embedding, this study demonstrated that basal cells derived from both murine and human sources can proliferate and form individual spherical structures that they termed “tracheospheres”. The tracheospheres possessed a P63-positive basal cell layer on the basal lateral side, and an inner apical lumen lined with Alpha-Acetylated-Tubulin-positive ciliated cells [6].

Airway organoids generated from both patient-derived basal cells and hPSCs can serve as powerful models for the study of disease mechanisms and possible drug therapies for airway respiratory illnesses. A prominent example of this is airway organoids that model cystic fibrosis (CF), a disease caused by mutations of a single gene—the cystic fibrosis transmembrane conductance regulator (CFTR) gene [72, 90]. So far, over 2000 disease-causing mutations of the CFTR gene have been identified. Accordingly, generating patient-specific organoids to screen for mutation-specific therapies that can restore CFTR function has been an important research milestone in this field [91, 92]. Further, airway organoids can also be used to model the defective cilia motility associated with primary ciliary dyskinesia (PCD) [32, 93, 94]. Airway organoids have also been used to model the plasticity of the airway epithelium in response to anti- and pro-inflammatory cytokines. For instance, the use of interleukin (IL)-13 and IL-6 has been shown to drive basal cell differentiation toward a secretory fate, and ciliated fate, respectively [95, 96]. Currently, there are no airway organoid models that can recapitulate mucociliary clearance, one of the primary functions of the conducting airway, suggesting that there remains much to be investigated in this field.

### Distal lung organoids

AEC2s have long been considered the resident adult stem cell population of the alveolus, possessing an established ability for self-renewal [18, 97]. While their capacity for differentiation to AEC1s are thought to be set in motion by injury, the mechanisms behind this are not well understood [31, 33, 82]. AEC2s isolated from mice were first accounted to generate alveolar organoids in 2013; FACS-enriched AEC2s cultured in an ALI/Matrigel system along with primary Pdgfrα-positive lung fibroblasts were shown to form 3D spherical structures termed “alveolospheres” comprising Sftpc-positive AEC2s and cells positive for AEC1 markers such as Aqp5 and Hopx [97]. However, unlike basal cells in the conducting airway, the epithelial population of the alveoli are far less accessible. Alternatively, as reported earlier, alveolar organoids can also be derived from hPSCs [14, 31, 98].

Alveolar organoids generated from both primary sources and hPSCs have demonstrated great potential to recapitulate disease pathology and address the shortage of physiologically relevant in vitro systems for therapeutic testing. An important example of this was the recent use of lung organoids as a model for severe acute respiratory syndrome coronavirus 2 (SARS-CoV-2) infection [99–101]. One such study demonstrated that the AEC2-like cells in the hPSC-derived lung organoids expressed the angiotensin-converting enzyme 2 (ACE2) receptor and are susceptible to SARS-CoV-2 infection, much like their native counterpart [99]. This model, to a certain extent, was able to recapitulate the inflammatory response that occurs in human lungs during SARS-CoV-2 infection. Additionally, they were also able to screen for several FDA-approved drugs that target the inhibition of SARS-CoV-2 entry. Further, hPSC-derived alveolar organoids have also been used to demonstrate the potential of gene editing tools such as CRISPR, which was recently used to correct a SFTPB mutation [31]. Despite the great advances in engineering lung organoids, vascularizing an hPSC-derived distal lung organoid to accurately recapitulate gas exchange in the alveoli remains an elusive goal in this field. However, a recent study tri-cultured normal lung fibroblasts, AEC2-like A549 cells, and endothelial cells to generate a first of its kind vascularized alveolar model, inching us a step closer [102].

### Mesenchymal participation in lung organoids

The pulmonary mesenchyme comprises myofibroblasts, smooth muscle cells, endothelial cells, and macrophages. [103, 104]. A seminal protocol recently described the derivation of both, mesenchymal and epithelial compartments in an hPSC-derived lung organoid [82]. These hPSC-derived “lung bud organoids” notably possessed heavily branched structures lined with both proximal and distal cell types, exhibiting tissue morphology and gene expression consistent with the developing human lung. More recently, a highly branched bronchioalveolar organoid (BALO) was established by 3D culturing FACS-enriched murine bronchioalveolar stem cells with resident fibroblasts and macrophages [10]. Single-cell sequencing confirmed that the cellular makeup of BALO was comparable to that of the native bronchioalveolar compartment in mouse lung. There are a few other co-culture systems that highlight the benefits of mesenchyme involvement. For example, an hPSC-derived cardio-pulmonary co-differentiated organoid system strikingly found expedited alveolar maturation in the company of cardiac mesoderm [34]. Further, hPSC-derived lung cells cultured with M1 and M2 macrophages to model inflammatory responses during SARS-COV-2 infection lead to the discovery of an interesting potential therapeutic strategy for COVID-19 involving M2 macrophages [105]. Research on other organ systems have also drawn on the idea of leveraging mesenchymal cells to generate more functional organoids. For instance, it was reported that the incorporation of human mesenchymal stem cells (hMSCs) can drive vascularization in hPSC-derived liver tissues [106]. The benefits of hMSC-driven self-condensation was later demonstrated in other organ models as well, including intestine, heart, kidney, brain, and especially the lung [107].

The pulmonary mesenchyme has been reported as the source of a complex signaling cascade regulating several key developmental events, including respiratory lineage specification, branching morphogenesis, and epithelial differentiation [103, 104]. The contributions from the pulmonary mesenchyme are so important, that strategies utilizing mesenchyme-derived growth factors, small molecule regulators of these growth factor signaling, or direct cellular co-culture have been developed to mimic this interaction during in vitro stem cell differentiation. Additionally, research on many pulmonary diseases such as asthma, which specifically target the mesenchymal compartment of the conducting airway, the airway smooth muscle, would benefit greatly from an in vitro multi-lineage organoid model [108]. While participation from non-epithelial pulmonary cell types is noticeably rare in most current lung organoid systems, this leaves much to look forward to in this field.

## Engineering cell–matrix interactions to optimize the in vitro stem cell niche

In the era of multi-omics, our understanding of the lung extracellular matrix (ECM) and its composition is continuously evolving. The lung ECM broadly comprises a network of collagen and elastin fibers enriched with proteoglycans (PGs), glycosaminoglycans (GAGs), and fibronectin [109].

It is immediately obvious that the ECM provides an elaborate framework that maintains structural integrity during lung organogenesis. However, it also plays a critical role in defining stem cell fate and maturation of the lung [20, 110]. The pulmonary ECM is a nexus of biochemical and mechanical cues that instructs stem cell behavior during lung development and regeneration [111]. For instance, proteoglycans found in the basement membrane ECM mediates FGF10 signaling that directs branching point specification during branching morphogenesis of rodent lungs [112, 113]. Similarly, the deposition of the structural protein elastin, especially in areas of the alveolus that are points for future alveolar crests is a driving force for secondary septation that occurs during alveolarization of the developing lung [114]. Laminin alpha5, an important component of the alveolar basement membrane also plays a critical role in directing alveolar epithelial cell maturation in the developing murine lung [115]. Inspired by native ECM, several attempts have been made to engineer matrices for in vitro stem cell maintenance and directed differentiation into pulmonary lineage. For instance, conditioned medium secreted by 804G cells is a popular source of laminin and collagen for in vitro expansion of airway basal stem cells [116, 117]. Additionally, Matrigel, a commercially available product comprising laminin, collagen IV and heparan sulfate proteoglycans isolated from Engelbreth-Holm-Swarm (EHS) sarcoma is commonly used either as a coating in 2D culture or as a hydrogel scaffold in 3D culture to direct stem cell differentiation into alveolar and airway epithelial lineages [31, 60, 118]. Alternatively, a 3D matrix of collagen I can also be applied to facilitate stem cell differentiation into multiple pulmonary epithelial lineages [29].

The composition of ECM also dictates its stiffness; proteins such as collagen and fibronectin influence tensile strength while elastin accounts for elastic recoil [119]. ECM stiffness has been shown to regulate stem cell differentiation through mechanosensing [120]. This is observed during murine lung development as the basement membrane is thinner at the epithelial buds than the surrounding ECM, generating a stiffness gradient that ultimately directs branching morphogenesis [121, 122]. While the lung stem cell field is due for more comprehensive studies exploring the effects of substate stiffness and matrix composition on in vitro hPSC differentiation into the pulmonary lineage, one recent study that investigated in vitro lung progenitor specification on two-dimensional (2D) Gelatin and Matrigel vs 3D Matrigel hinted at a preference for the less-stiff 3D Matrigel substrate [46]. There has also been a special focus on engineering lung ECM-specific hydrogels to recapitulate the stiffness and composition of the native lung [123, 124].

Advances in biomaterial engineering and fabrication also presents the potential for further improvement in recapitulating cell–matrix interactions and organotypic tissue architectures of the lung. Whole-organ decellularization provides a platform for lung cell engraftment in lung-specific ECM and spatial specific cell seeding into the alveolar and vascular compartments [125–128]. Technology developments in additive manufacturing have also facilitated the fabrication of ECM scaffolds that more closely recapitulate native lung architecture. Specifically, solubilized bioinks prepared from purified or decellularized ECM materials can potentially be 3D printed alongside desired cell populations to generate scaffolds or cellular tissue grafts of desired geometries [129–131]. Moreover, signaling molecules, such as growth factors and glycosaminoglycans (GAGs), can be incorporated to further functionalize ECM biomaterials via enzymatic or chemoselective approaches to generate a signaling reservoir to modulate cellular differentiation and tissue morphogenesis [129, 132]. One of the greatest challenges lies in achieving temporal and spatial specific delivery of signaling molecules with high resolution, like during branching morphogenesis of the native lung, when the ECM establishes a unique pattern for Fgf10 signaling gradient via differential Fgf10 expression of mesenchymal cells, promoting branching morphogenesis (Fig. 2). While some initial attempts have shown the possibility to achieve spatial-specific biomaterial functionalization (Fig. 2b) [133], their eventual implementation in engineering complex tissues such as the lung will require extensive future advancements from both scientific and engineering investigations.

A deeper understanding of ECM dynamics accompanying lung cell induction and tissue formation offers the promise to further boost our ability to modulate cell-ECM interactions for directed lung tissue morphogenesis. The ECM microarray technique enables high-throughput screening of multiple combinations of ECM components by generating thousands of different artificial niches for testing stem cell responses [134]. With the rapid development of mass spectrometry, the proteomic analysis of stem cell niche starts to contribute to the identification of novel proteins from the matrisome (ECM and ECM-associated proteome) that promote stem cell differentiation. With higher resolution and sensitivity in characterizing matrisome dynamics, key ECM factors may be discovered for optimizing the extracellular stem cell niche in vitro.

## Concluding remarks

Lung development is a complex but highly coordinated process regulated by a considerable number of signals and pathways. These signals are produced and secreted by cells in the epithelium and mesenchyme, serving as cues to modulate lineage-specific gene expression, guide cell migration, and specify cell fates.

As discussed earlier, many seminal attempts have been made to direct hPSCs differentiation in vitro by recapitulating key programs of native lung development [27, 31–33, 72]. Many of these protocols are based on inducing the signals found in vivo in a temporally specific, stepwise manner. While these innovative efforts cannot be understated, there is still more to be investigated. For instance, very little is understood about directing in vitro hPSC differentiation into AEC1, a cell type that facilitates the most prominent function of the lungs, gas exchange. Generally, features such as gene expression, morphology, and functionality are assessed in combination to identify cells in vitro*.* The lack of an established functionality assay also potentially hinders the optimization of hPSC differentiation into AEC1. Additionally, the goal of interfacing tissue engineered lung epithelium with a perfusable vascular component remains an area of active exploration.

In vivo lung stem cells behaviors are highly regulated by their microenvironments, comprising cell–cell interactions and cell-ECM interactions. Several multi-lineage co-cultures and ECM systems have been established to improve the biomimicry of in vitro stem cell models. However, there remains a critical need to expand our understanding of these interactions during both native and engineered lung morphogenesis to improve our ability to engineer lung stem cell niches. For instance, characterizing cell type-specific secretome in multi-lineage culture systems can reveal how cells interact with one another. Additionally, studying ECM dynamics during native lung organogenesis can lead to the discovery of novel extracellular factors that can promote stem cell differentiation or their integration into higher order tissue structures.

## Data Availability

Not applicable.

## Abbreviations

hPSCs: Human pluripotent stem cells
ECM: Extracellular matrix
AEC1: Type 1 alveolar epithelial cells
AEC2: Type 2 alveolar epithelial cells
BASCs: Bronchio-alveolar stem cells
ESCs: Embryonic stem cells
iPSCs: Induced pluripotent stem cells
TGF-β: Transforming Growth Factor-β
DE: Definitive endoderm
AP: Anterior–posterior
DV: Dorsal–ventral
PD: Proximal–distal
Bmp4: Bone morphogenetic protein 4
RA: Retinoic acid
Fgf4: Fibroblast growth factor 4
AFE: Anterior foregut endoderm
Dkk1: Dickkopf-related protein 1
EGF: Epidermal Growth Factor
KGF: Keratinocyte Growth Factor
cAMP: Cyclic adenosine monophosphate
FACS: Fluorescence activated cell sorting
3D: Three-dimensional
2D: Two-dimensional
BALO: Bronchioalveolar organoid
MAPK: Mitogen-activated protein kinase
ALI: Air–liquid interface
IL: Interleukin
CFTR: Cystic fibrosis transmembrane conductance regulator
PCD: Primary ciliary dyskinesia
PGs: Proteoglycans
GAGs: Glycosaminoglycans
EHS: Engelbreth-Holm-Swarm
